# *Plasmodium vivax* and *Plasmodium falciparum ex vivo* susceptibility to anti-malarials and gene characterization in Rondônia, West Amazon, Brazil

**DOI:** 10.1186/1475-2875-13-73

**Published:** 2014-02-28

**Authors:** Anna Caroline C Aguiar, Dhelio B Pereira, Nayra S Amaral, Luiz De Marco, Antoniana U Krettli

**Affiliations:** 1 Laboratório de Malária, Centro de Pesquisas René Rachou, FIOCRUZ, Av. Augusto de Lima 1715, 30190-002 Belo Horizonte, MG, Brazil; 2 Faculdade de Medicina, Universidade Federal de Minas Gerais, Belo Horizonte, MG, Brazil; 3 Centro de Pesquisas em Medicina Tropical de Rondônia, Porto Velho, Rondônia, Brazil

**Keywords:** Anti-malarials, Resistance, *Plasmodium vivax*, *Plasmodium falciparum*, Chloroquine resistance

## Abstract

**Background:**

Chloroquine (CQ), a cost effective antimalarial drug with a relatively good safety profile and therapeutic index, is no longer used by itself to treat patients with *Plasmodium falciparum* due to CQ-resistant strains. *P. vivax*, representing over 90% of malaria cases in Brazil, despite reported resistance, is treated with CQ as well as with primaquine to block malaria transmission and avoid late *P. vivax* malaria relapses. Resistance to CQ and other antimalarial drugs influences malaria control, thus monitoring resistance phenotype by parasite genotyping is helpful in endemic areas.

**Methods:**

A total of 47 *P. vivax* and nine *P. falciparum* fresh isolates were genetically characterized and tested for CQ, mefloquine (MQ) and artesunate (ART) susceptibility *in vitro*. The genes *mdr1* and *pfcrt*, likely related to CQ resistance, were analyzed in all isolates. Drug susceptibility was determined using short-term parasite cultures of ring stages for 48 to 72 hour and thick blood smears counts. Each parasite isolate was tested with the antimalarials to measure the geometric mean of 50% inhibitory concentration.

**Results:**

The low numbers of *P. falciparum* isolates reflect the species prevalence in Brazil; most displayed low sensitivity to CQ (IC50 70 nM). However, CQ resistance was rare among *P. vivax* isolates (IC_50_ of 32 nM). The majority of *P. vivax* and *P. falciparum* isolates were sensitive to ART and MQ. One hundred percent of *P. falciparum* isolates carried non-synonymous mutations in the pfmdr1 gene in codons 184, 1042 and 1246, 84% in codons 1034 and none in codon 86, a well-known resistance mutation. For the *pfcrt* gene, mutations were observed in codons 72 and 76 in all *P. falciparum* isolates. One *P. falciparum* isolate from Angola, Africa, showing sensitivity to the antimalarials, presented no mutations. In *P. vivax*, mutations of *pvmdr1* and the multidrug resistance gene 1 marker at codon F976 were absent.

**Conclusion:**

All *P. falciparum* Brazilian isolates showed CQ resistance and presented non-synonymous mutations in *pfmdr1* and *pfcrt*. CQ resistant genotypes were not present among *P. vivax* isolates and the IC_50_ values were low in all samples of the Brazilian West Amazon.

## Background

Malaria, one of the most prevalent parasitic diseases in the world, still causes high morbidity and death, mainly in *Plasmodium falciparum*-infected, non-treated patients [1]. *Plasmodium vivax* causes intense morbidity and contributes to significant political, social and economic instability in developing countries of Latin America and Asia [2,3]. CQ is the drug of choice to treat *vivax* malaria in endemic areas of Brazil and primaquine (PQ) is used to avoid late malaria relapses [3]. The recommended dose for adults is 1500 mg of CQ (daily for three days) and 210 mg of PQ (daily for seven days) [4]. *Plasmodium vivax* resistance is now widespread and has rendered CQ ineffective in parts of Indonesia and Papua New Guinea [5-7]. Low levels of resistance have also been reported in Myanmar, South Korea, Vietnam, India, Turkey, Ethiopia, and in regions of Southern Africa and South America [3,8,9]. The occurrence of severe *vivax* malaria and patient’s deaths has been reported in Brazil [10-12] raising the possibility of an association between malaria severity and drug resistance [13]. In areas of CQ resistance, treatment of uncomplicated *P. falciparum* malaria is carried out with artemisinin-based combination therapy (ACT) [3]. Drugs that complement ACT include lumefantrine, amodiaquine, AQ, MQ, sulphadoxine-pyrimethamine and antibiotics. In Brazil, the first option for *falciparum* malaria treatment is the combination of artemether (480 mg daily for four days) and lumefantrine (2880 mg daily for four days). PQ (45 mg) is administrated on day one to avoid malaria transmission. These doses are recommended for adults with 50 Kg weight or more [4]. A reduced susceptibility to artemisinin derivatives has been described in *P. falciparum*-treated patients [14,15].

Increasing evidence of a lower *P. vivax* susceptibility to CQ in malaria-endemic areas [16] includes the state of Amazonas [17] and is believed to be associated with malaria’s clinical severity [18].

Molecular markers associated with CQ resistance are non-synonymous mutations in the drug/metabolite transporter gene *pfcrt* (C72S, K76T) and in the multidrug resistance protein 1 gene *pfmdr1* (N86Y; Y184F; S1034C; N1042D; D1246Y), described in *P. falciparum*[19-21]. One mutation of the *multidrug resistance gene 1* (Y976F) of *P. vivax* is also associated with parasite susceptibility to CQ [8]. A non-synonymous mutation of the *pvdhps* gene at codon 382 (S382C) was recently associated with *in vitro* susceptibility to CQ [18]. The present study aimed to examine the phenotypic and genotypic chemoresistance profile of *P. falciparum* and *P. vivax* to commonly used anti-malarial drugs in a Brazilian malaria-endemic area in the Amazon Region.

## Methods

### Subjects

**Figure 1:**
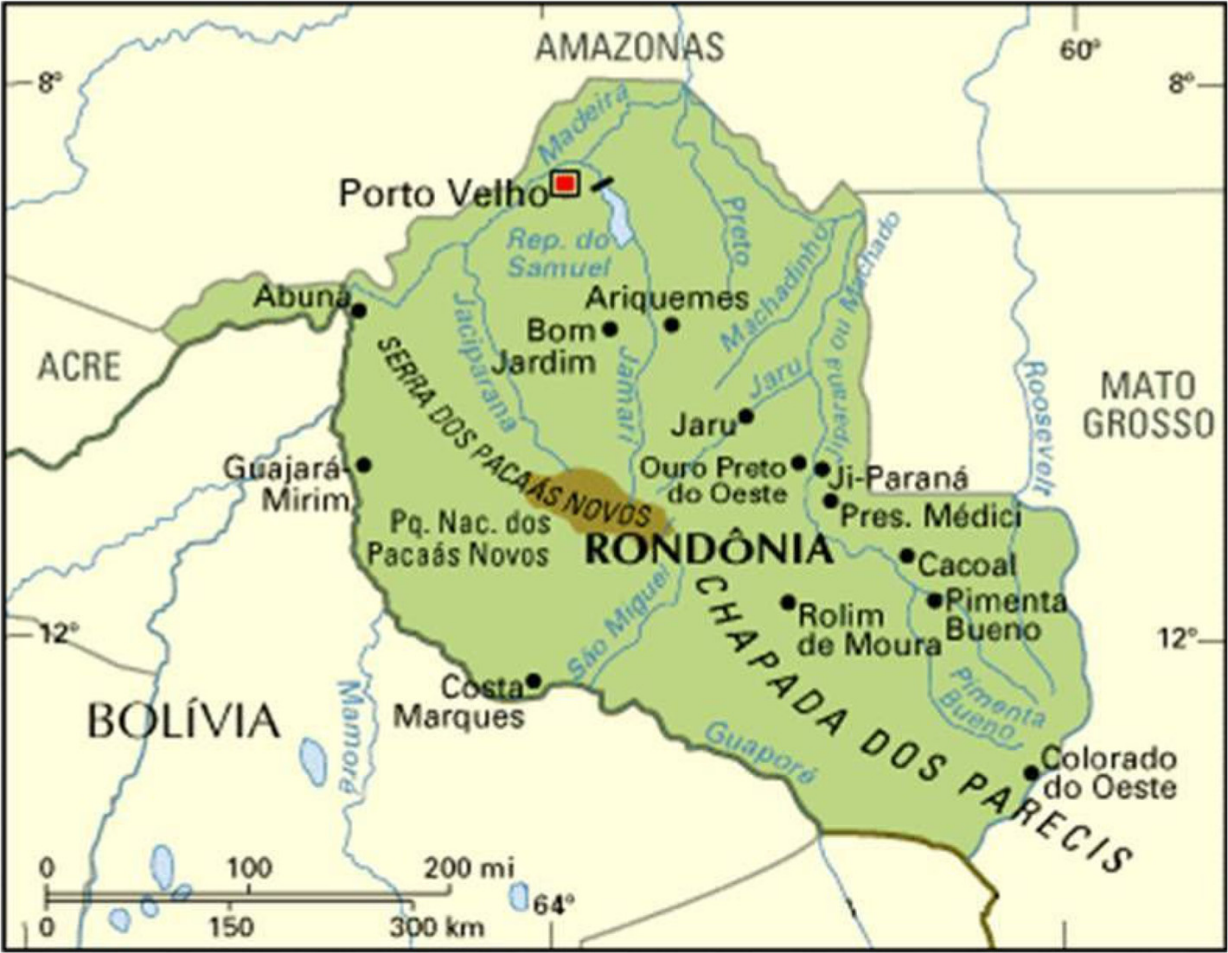
Rondônia state, West Amazon.

All isolates were collected between August 2012 and March 2013 from patients recruited at the Centre of Malaria Control (CEPEM) in the city of Porto Velho, state of Rondônia, in the Brazilian Western Amazon, where *P. vivax* is highly prevalent. Only patients mono-infected with either *P. falciparum* or *P. vivax* and with high parasitaemia (between 2,000 and 80,000 parasites/μl) were recruited. Patients who used any anti-malarial in the previous month and/or presented severe symptoms of malaria were excluded from this work. The study cohort encompassed 56 patients living in this highly endemic area which is close to Bolivia (Figure 1). Forty seven patients were diagnosed with *P. vivax* and eight with *P. falciparum*. In addition, one isolate of an individual infected with *P. falciparum* with imported malaria (from Africa) was also studied. One patient had mixed malaria (*P. vivax* and *P. falciparum*) and was not included. From each volunteer, a peripheral venous blood sample (5 ml) was collected by venipuncture in heparin-containing tubes and immediately used for the *ex vivo* drug susceptibility assay using pre-prepared plates with the diluted anti-malarials, as described below. DNA was also extracted from peripheral venous blood in EDTA containing tubes for parasite genomic analysis.

### Ethical approval

This study was approved by the Ethics Committee, Centro de Pesquisas René Rachou-FIOCRUZ (CAAE -03209212.7.0000.5091). All participants signed a written informed consent before blood collection.

### Pre-dosed plates with test and control drugs

CQ, MQ and ART were prepared as 10 μg/mL stock solution in dimethyl sulphoxide (DMSO) in 96-well plates (20 μL per well), then diluted two-fold in RPMI, with variable maximum drug concentration according to each previously determined activity (shown in parentheses), i.e. CQ (854 nM), MQ (724 nM), ART (738 nM), lyophilized and stored at 4°C until further use. Each lot of plates was assayed for quality control just before use, based on the profile response to CQ to the *P. falciparum* laboratory strains W2 and 3D7, for CQ-resistance and -sensitivity, respectively. All plates were prepared in our laboratory, in Belo-Horizonte, transported to the field in dry ice and kept at minus 70°C until use.

### *Ex vivo* drug susceptibility assay

The drug susceptibility of the malaria parasites, from each patient, was measured *in vitro*. For *P. falciparum* the method used was described by Rieckmann and Antuñano [22] and for *P. vivax* by Renapurkar *et al*. [23] with modifications [24]. White blood cells were removed by filtration in a CF 11 cellulose column as described [25]. Immediately before the *ex vivo* drug susceptibility assays, packed red blood cells with the parasites (iRBC) were diluted for a 2% hematocrit, using either complete medium RPMI 1640 medium plus 10% AB human serum, with *P. falciparum* cultures; or the McCoy’s 5A medium plus 20% AB human serum, with *P. vivax* samples. The iRBC (200 μl per well) were distributed in the pre-dosed drug plate. For the maturation of parasites, rings to schizonts, the plates were maintained in candle jars at 37°C as described [26], at different incubation times (48–72 hours). The control wells were iRBCs and cultured with drug free complete medium. The incubation parasite-drug was stopped when 40% of the ring stages reached the schizont stage (at least four distinct nuclei per parasite) in the drug-free control wells (n = 6 per plate). Thick blood films were then made from each well, dried, stained with 5% Giemsa solution for 30 min, and examined microscopically. The number of schizonts per 100 asexual stage parasites was determined for each drug concentration and then normalized by comparing with the schizont number in the drug-free control wells (considered as 100%). The half-maximal drug inhibitory response (IC_50_) was estimated by curve fitting using software (OriginLab Corporation, Northampton, MA, USA) and comparing with parasite growth in the drug-free controls.

### Real time PCR for *Plasmodium* detection

Genomic DNA of the parasites was extracted using QIAamp DNA kit (QIAGEN, Chatsworth, CA, USA) and then subjected to real time PCR (Applied Biosystems®). The 18S rRNA gene was chosen as target gene since it contains both highly conserved and variable regions (at least five copies of the gene are dispersed on separate chromosomes of the *Plasmodium* genome). Each 20 μl reaction mix contained 2 μl of sample DNA, 10 μl FastSTART DNA SYBR Green reagent (Roche), 6.5 mM MgCl2 (final concentration), and 0.5 mM concentrations of each primer (5′-TAACGAACGAGATCTTAA-3′and 5′-GTTCCTCTAAGAAGCTTT-3′). The PCR conditions consisted of an initial denaturation at 95°C for 10 min, followed by amplification for 40 cycles of 10 sec at 95°C, 5 sec at 50°C, and 20 sec at 72°C, with fluorescence acquisition at the end of each extension step. Amplification was immediately followed by a melt programme, consisting of 2 min at 95°C, 2 min at 68°C, and a stepwise temperature increase of 0.2°C/sec until 90°C, with fluorescence acquisition at each temperature transition. Fluorescence was analysed using F1/F2 settings, which improved the detection of *P. falciparum* (a cutoff of 35 cycles was used to define *Plasmodium*-positive samples). A melting curve analysis was used to determine the species-specific mean melting temperature (Tm) based on values determined from the respective positive controls [27].

### Analysis of the *crt* and *mdr1* genes

The *pfcrt* and *mdr1* genes were amplified by PCR with specific primers for each region (Table 1).The PCR reactions were performed with 3 μl of DNA at 30 ng/μl, mixed with 6.5 μl of AmpliTaq Gold® PCR Master Mix (Applied Biosystems, Warrington, UK), 0.5 μl of each primer at 10 pmol/μl, on a final volume of 15 μl. The samples were placed in an Eppendorf Mastercycler® (Hamburg, Germany) at 94°C for 3 min followed by 35 cycles of 94°C for 30 sec, 55°C for 30 sec and 72°C for 30 sec and a final extension time at 72°C for 5 min. PCR products were purified using Wizard PCR DNA and Gel Band Purification Kit (Promega, Madison, USA) following manufacturer’s protocol and visualized on a silver-stained 6.5% polyacrylamide gel. Purified DNA fragments were then sequenced using the dideoxy method. Sequence data were analysed using Sequencher 4.9 software (Gene Codes Co, Ann Arbor, MI, USA). Sequences were then compared with those on Plasmo DB gene bank.

**Table 1:** **Set of primer sequences used to characterize gene polymorphisms by** ***Plasmodium falciparum*** **and** ***P*.** ***vivax***

**Gene**	**Codons**	**Primer**	AT* (°C)	**PCR products (bp)**
*pfmdr1*	86, 184	5′-GAGTTGAACAAAAAGAGTACCGCTGA-3′	55	512
		5′-TTTTTCCGTTAATTTATGTTTGTGGTGTCA-3′		
	1043, 1042	5′-TGTCAAGCGGAGTTTTTGCATTTAGT-3′		299
		5′-TGGTAGTTATGCTGGAAAATTAATGTCCT-3′		
	1246	5′-GGAGAAACAGGTAGTGGAAAATCAACTT-3′		302
		5′-TTTGGAAGAGAAGATGCAACATTGGAA-3		
*pvmdr1*	976	5′-ACTCACTTTATAGTGCTCTTCCTTGTG-3′	55	476
		5′- GGACATCAACTTCCCGGCGT- 3′		
*pfcrt*	72, 76	5′- acagATGGCTCACGTTTAGG -3′	55	162
		5′- TTTTGTAACATCCGAAACTCACA -3′		

*Annealing temperature.

## Results

### Anti-malarial susceptibility

*Ex*-*vivo* drug susceptibility was assessed in field isolates from all 56 patients with a single species infection, either *P. vivax* (n = 47) or *P. falciparum* (n = 9); eight of the latter were Brazilian isolates freshly collected in the state of Rondônia, in the Brazilian Western Amazon (Figure 1) and one was from an individual returning from Angola, Africa, diagnosed and studied in Belo Horizonte, Brazil, outside the endemic malaria region.

Adequate parasite growth was achieved in 100% (9/9) of the *P. falciparum* and in 68% (32/47) of *P. vivax* isolates. The characteristics of these isolates are summarized in Table 2. The majority of *P. falciparum* Brazilian isolates studied were considered resistant to CQ (IC_50_ 70 nM; range, 0.19 to 223 nM); two susceptible showed IC_50_ values of 0.19 and 37 nM; two isolates were considered resistant to MQ (IC_50_ of 50 and 63 nM); but, all isolates were susceptible to ART, since the highest IC_50_ was 5.8 nM.

**Table 2:** Characteristics of the study population with malaria in the Amazon-Brazil

	***P*. *falciparum***	***P*. *vivax***
**Number of patients**	**9**	**47**
Male (age)	N = 6 (41 ± 4)	N = 37 (37 ± 10)
Female (age)	N = 3 (22 ± 3)	N = 10 (47 ± 13)
Fever at the time of blood collection	9	42

Among the *P. vivax* isolates the results of drug susceptibility to CQ suggests no resistance; the median IC_50_ was 32 nM; ranging between 3 to 69 nM (Table 3); four isolates (P010, P024, P026, P042) less susceptible to CQ showed IC_50_ values higher than 51 nM. In further analysis 32 were examined in the thick smears for the stages of the parasite blood forms; three patients had approximately 30% of parasites in trophozoite stage, which is considered a high rate. And, they present high IC_50_ values to ART and MQ (Table 4). However this number is not enough to show a strong association between stage-specificity and drug activities and future studies are needed. The only African isolate studied was susceptible to the anti-malarials tested (Table 3).

**Table 3:** **Overall** ***ex*-*vivo*** **sensitivity of Brazilian isolates for each drug, according to the species tested, and for laboratory** ***P*.** ***falciparum*** **(W2 clone) line, chloroquine resistant**

**Drug**	***P*. *falciparum***					***P*. *vivax *** **Clinical field isolates**	
	**W2**	Fc27*	***African***	**Clinical field isolates**			
	**IC**_**50 **_**(nM)**			**n**	**Median IC**_**50 **_(nM)**	**n**	**Median IC**_**50 **_**(nM)**
Chloroquine	178	39	19	9	70 (0.19-223; 80)		32 (3–69; 64)
Artesunate	18	16	1.5	9	1.3 (0.26-5.8; 2.82)	32	21 (0.08-137; 17.4)
Mefloquine	19	11	46	9	21 (1.19-63; 80)		57 (5.0-113; 82)

*Date from Marfurt et al. [28,29]. **The numbers inside of the parenthesis means: lowest IC_50_, higher IC_50_ and interquartile range.

**Table 4:** **Anti-malarial activity** ***in vitro*** **in 15 different human** ***P*.** ***vivax*** **isolates for chloroquine (CQ), artesunate (ART) and mefloquine MQ)**

***P*. *vivax*** **isolates**	**% of trophozoites**	**Drugs/IC**_**50 **_**Nm**		
		**CQ**	**ART**	**MQ**
P006	22	29	15	11
P010	20	61**	11	45
P015	9	13.5	11	6
P022	15	8	11	60
P024	32*	69**	34	113
P025	93*	3.0	28	11
P026	29	51**	137	42
P028	11	6.6	2.8	11
P029	16	34	2.5	5.5
P031	10	15	8.0	8.2
P033	8	23	23	24
P034	17	29	9.6	47
P038	42*	8.5	1.3	2
P037	32*	6.5	9.4	39
P039	88*	6.8	5.2	26
P042	80*	62**	23	15
Mean (±SD)	-	23 + 9	32 + 37	32 + 28

*Isolates that presented more than 30% trophozoites. **Isolates that were considered less sensitive to CQ.

### Characterization of the *CRT* and *MDR1* resistance

The parasite profiles of molecular resistance for *P. vivax* isolates, using the frequencies of SNPs (Single Nucleotide Polymorphism) in *pvmdr1* codon 976, were examined in 47 samples (Table 1). The product was compared with the genomic sequence of Salvador I as the reference wild type found in the Plasmo DB gene bank, and no SNPs were found. For *P. falciparum* isolates, frequencies of SNPs in *pfmdr1* codons 86, 184, 1034, 1042 and 1246 are shown in Table 5. In addition, codons 72 and 76 of *pfcrt* gene were also evaluated (Table 5). Mutant alleles at positions 184, 1042 and 1246 on pfmdr1 gene were present in 100% of the samples. In codon 1034, the frequency of mutation was 84% and no mutations were found in codon 86. The *pfcrt* gene carried SNPs in codons 72 and 76 in all Brazilian isolates. All isolates presented non-synonymous mutations (Table 5). The *P. falciparum* isolate from outside Brazil (Angola, Africa) had no SNPs on the *pfcrt* or *pfmdr1* genes.

**Table 5:** **Prevalence of molecular markers associated with** ***P*.** ***falciparum*** **resistance to chloroquine**

**Gene – codon**	**% of mutations**	**Mutations**	**Protein change**
*PfMDR1*			
86	0%	-	-
184	100%	TAT **>** TTT	Tyrosine **>** Phenylalanine
1034	84%	AGT **>** TGT	Serine **>** cysteine
1042	100%	AAT **>** GAT	Asparagine **>** Aspartic acid
1246	100%	GAT **>** TAT	Aspartic acid **>** Tyrosine
*PfCRT*			
72	100%	AAA **>** ACA	Lysine **>** Treonine
76	100%	TGT **>** TCT	Cysteine **>** Serine

## Discussion

Due to the strong impact of chemo-resistance among the malaria parasites to most drugs used in the control of the disease, monitoring the development of resistant phenotypes and genotyping are priorities wherever endemic malaria is present. The *in vitro* methods used to examine anti-malarial drug sensitivity provide a profile of *Plasmodium* sensitivity to a variety of drugs, simultaneously assayed. However, a lower *in vitro* sensitivity of a parasite isolate does not imply drug-resistance *in vivo*, as other factors can interfere, which are not determined by *in vitro* tests. The *ex vivo* tests provide an outline of resistant-circulating phenotypes for each tested drug, provided that an adequate number of patients are examined in a given area. This was not the case for *P. falciparum* in our study: only nine patients were evaluated, reflecting the reduced transmission of this parasite species in Brazil [30]. Determining parasite genotype, performed in parallel, provides further information since mutations in the *pfcrt* gene alter the CQ flux and/or reduce drug efficacy [31]; and provides data for policy makers to decide the best drug to be prescribed in that area. Ideally, monitoring anti-malarial chemo-resistance must be continuous since development and spreading of resistance are dynamic events, changing with time and according to human interventions and other factors such as population migration [32].

The antiplasmodial activity and the molecular profile of resistance by anti-malarial standards like CQ, ART and MQ, confirms *P. falciparum* resistance to CQ only, as demonstrated in *ex*-*vivo* tests against *P. vivax* and *P. falciparum* isolates from patients with naturally acquired malaria in the state of Rondônia in the Brazilian Western Amazon, which is substantiated by sequencing of genes related to resistance to anti-malarial drugs.

There is evidence of *P. vivax* resistance to CQ in the state of Amazonas, specifically in the city of Manaus where an increase in the proportion of *P. vivax* malaria parallels an increase of unusual clinical complications related to this species [33]. These authors used an *in vivo* test to assess the efficacy of standard supervised CQ therapy. Among 109 volunteers with *P. vivax* who completed the *in vivo* test, 19 had positive blood smears within the 28-days follow-up (one on day 14, three on day 21, and 15 on day 28). All were then required to undergo an alternative therapy with MQ. In another study performed by the same group, a lower *P. vivax* susceptibility to CQ through the attainment of IC_50_ by ELISA assay or using traditional methods [18,34] was examined; they demonstrated that drug-resistance was related to the presence of non-synonymous mutation at *pvdhps*, *pvcrt* and *pvmdr1*.

All *P. vivax* isolates presently studied in the Brazilian Western Amazon were sensitive to CQ in *ex vivo* assays. Although the threshold of IC_50_ to define a sample as resistant to CQ is not well established for *P. vivax*, it has been proposed that the same threshold used for *P. falciparum* should be used, with 100 nM threshold of CQ [35].

The IC_50_ for ART and MQ in *P. vivax* were also examined and they were higher when compared with the values found in *P. falciparum*. It can be due to differences in the stage-specific activities of CQ, MQ and AQ in *P. vivax*, demonstrated here and by Marfut *et al*. [28]. It may be interesting to compare the susceptibility of *P. vivax* in strains from other regions of the world. This issue remains to be further studied using strains from different places.

Only one point mutation for CQ was studied for *P. vivax* in the *pvmdr1* gene codon 976, but other genes were associated with CQ resistance in the Brazilian Amazon, e.g., in the pvdhps gene in codon 382 (S → C) [18].

Considering that the severity of *P. vivax* malaria in the state of Amazonas has been attributed to CQ resistance and to the increased levels of *pvmdr1* and *pvcrt*-*o* compared to the levels expressed by parasites from patients with mild symptoms [36], these genes copy number could also be evaluated. In addition, the *mdr1* copy number is strongly associated with recrudescence after artesunate-mefloquine administration, and could be used as a surveillance tool for artesunate-mefloquine resistance, as reported in patients in Cambodia [37,38].

In conclusion, in West Amazon, most *P. falciparum* isolates were CQ resistance, a data confirmed by parasite genotyping. No mutations were found for *P. vivax* in the region supporting the lower prevalence of this strain in Brazil.

## Competing interests

The authors declare that they have no competing interests.

## Authors’ contributions

ACCA and NSA carried out the molecular studies. DBP was the MD who interviewed and treated the patients in the endemic area and ACCA performed the ex vivo and diagnostic exams tests. AUK and LDM conceived the studies, participated in the experimental design and were responsible for the biological tests. AUK was the project leader. All authors read and approved the final manuscript.
